# Utilizing the Momentum in Orbital Angular Momentum: Augmented OAM induced by a $\frac{{\boldsymbol{\pi }}}{{\bf{2}}}$ Aperture of Three Elements

**DOI:** 10.1038/s41598-018-34139-7

**Published:** 2018-10-23

**Authors:** Reham M. Fouda

**Affiliations:** RMIT University, School of Engineering, Melbourne, 3000 Australia

## Abstract

The feasibility to induce augmented dominant OAM modes by a *π*/2 aperture of three elements in space and weighted quasi-phase shifts is realised in this paper. It is shown through theory, numerical simulations and experimentation, that electromagnetic (EM) waves carrying non-integer OAM with dominant mode *l* = +1 in the microwave domain can be generated by a quarter of a full azimuthal annular aperture consisting of three elements and a weighted phase shift augmenting the expected conventional phase shift to reach Berry’s mode dominance theory of half integer *l*. With reference to the uncertainty principle of angular momentum and angular position, the proposed augmented OAM with weighted phase shift method seems to decrease mode uncertainties and augment mode dominance.

## Introduction

An exciting characteristic of Electromagnetic (EM) waves, recognised by Allen *et al*. in 1992, is their ability to carry Orbital Angular Momentum (OAM)^1^ of *lℏ* per photon, in addition to the spin ±*ℏ* component in angular momentum. Much attention has been given to OAM from the research community for a diverse number of applications within the Optical, Millimetre Wave, Microwave, and Radio frequencies regimes.This is due to the inherently orthogonal nature of OAM carrying waves, which can take on a theoretically unlimited number of orthogonal states^2^. This new degree of freedom using an EM wave’s orthogonality meant that information can be encoded on the same frequency^3^, alleviating spectral congestion and increasing channel capacity^4^ in communications. Other applications where OAM found influence is in Optical Tweezers^5^, Astrophysics^6^, and Radar Super-Resolution Imaging^7^.

OAM has particularly proven to be capable of innovating communication systems, whilst introducing a new degree of freedom across a large portion of the electromagnetic (EM) spectrum. In particular, within the optical regime, where divergence is minimal in contrast with lower frequencies. Optical multiplexed OAM links have shown to be capable of expansively increasing channel capacity for Terabit-Scale communications in free-space^8,9^ and optical fibers terabitF. Moreover, the distances reached for optical OAM-based communication links are well over the kilometer (km) range within free-space^10^ and optical fibers^11^. Further studies on OAM’s characteristics such as behaviours of fractional topological charges and transmission of fractional OAM beams has lead to applications within the extreme-ultraviolet^12^ and higher optical frequencies^13^.

The exploitation of OAM’s potential within the lower frequencies of the EM spectrum, however, is constrained by the need for large impractical apertures, and significant divergence^14^ due to longer wavelengths as compared to optical wavelengths. It is therefore of priority to investigate ways in which we can decrease the aperture size whilst preserving the ability to dynamically generate stable OAM modes. Several methods introduced to address the aforementioned challenges are compact planar phased arrays^15,16^, Metasurfaces^17^ for OAM mode generation and partial angular receiving apertures^18,19^. However, restraining the angular aperture would result in increased distribution of angular momentum states according to the uncertainty principle^20^, and hence; increased mode cross talk. OAM generated by restricted apertures also correspond to non-integer values of OAM, which are superpositions of integer *l* modes. The overall mode will therefore be the average of all these modes, corresponding to the generating aperture’s intended *l* mode only for integer and half-integer values of OAM^21^.

Another method proposed from previous work, a Quasi-Circular Array Antenna (QCA)^22^ for dynamic Quasi-OAM generation with a lower limit on the number of elements required on the aperture with respect to the inter-element angle *β*, ${N}_{ {\mathcal L} }\ge \frac{\pi }{\beta }$. This is to ensure a magnitude of phase shift |*δφ*| ≥ *lπ*, where the inter-element phase shift obeys the theoretical *δφ* = 2*πl*/*N* for an annular aperture with *N* number of elements^23^. In this paper, the feasibility to generate Quasi (non-integer) OAM beams with less elements than those constrained by half-integer apertures where the total phase shift would otherwise be |*δφ*| ≥ *π*, is demonstrated.

## Results

**Figure 1:**
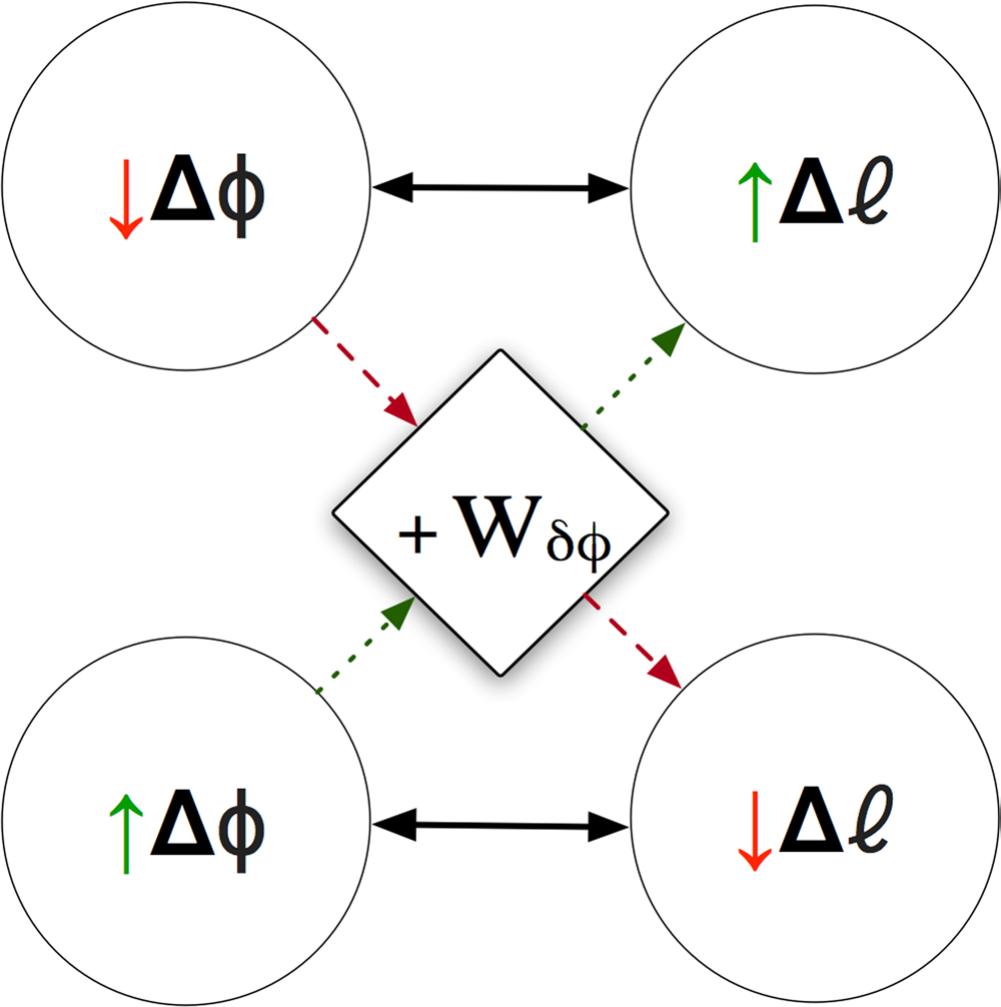
The effect of the weighted phase shift parameter *W*_*δφ*_ on the measured OAM mode uncertainties Δ*l* for restricted and unrestricted angular apertures Δ*ϕ*.

**Figure 2:**
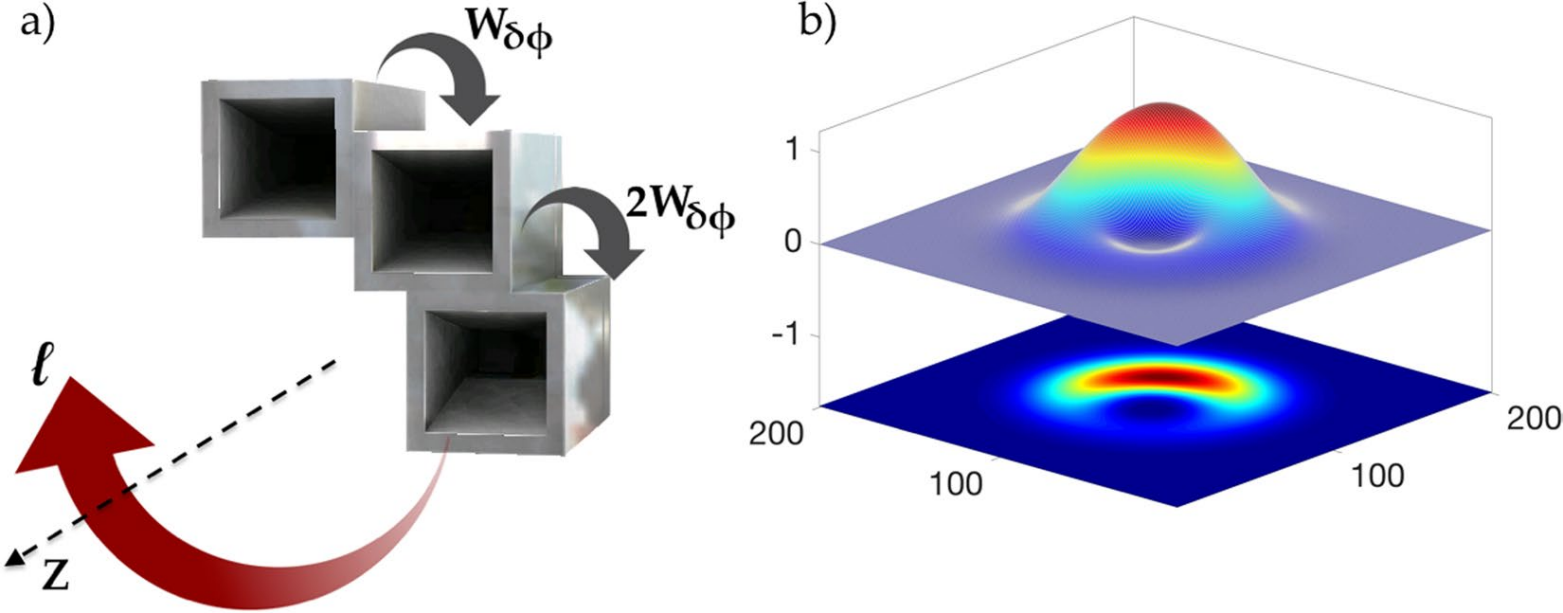
Three element quasi-circular array antenna (QCA) **(a)** in a restricted annular aperture of $\frac{\pi }{2}$ and a weighted phase shift *W*_*δφ*_ between each element. The numerically calculated beam direction and cross section **(b)** profile of the quasi-OAM mode *α* = 0.375.

A *π*/2 aperture consisting of three elements is proposed with a weighted phase shift to augment the total endured phase shift, satisfying Berry’s total non-integer OAM strength theory^24^ for intended *l* mode dominance. Phase weightings are introduced as another parameter in restricted apertures of *N* elements less than *π*, which is anticipated on the basis of the Aharonov-Bohm effect in quantum mechanics^25^ to stabilise the OAM intended mode with a weighted quasi phase shift less than 2*πl*. The results in this paper appear to show a relation, not only between angular position and angular momentum^20^ corresponding to the uncertainty principle in quantum theory^26^, but also a possible relation with the total endured phase shift |*δφ*|. It appears that an increase in the magnitude of endured phase shift from a restricted annular aperture corresponds to an increase in OAM mode dominance and hence; preciseness. This relationship can be described as in Fig. 1 and later demonstrated in simulation and experimental results. Consider a three element array (${N}_{ {\mathcal L} }=3$) as seen in Fig. 2(a) with an angular aperture of $\frac{\pi }{2}$ and inter-element distribution angle *β* = 45°. Each element is consecutively phase-shifted by +45° in accordance with the theoretical inter-element phase shift^23^ value given by Eq. (1) for OAM *l* = +1. In the case where the angular aperture is not restricted (ie; a whole circular array), the above phase configuration would result in a phase shift magnitude |*δφ*| = 360° (2*π*). Hence, a theoretically pure *l* mode.

1 $$\delta \phi =2\pi l/N$$

As we decrease the number of elements, the same theoretical phase shift according to Eq. (1) would result in a smaller magnitude in phase shift, proportional to the angular restriction. In the case of ${N}_{ {\mathcal L} }=3$ elements, and *δφ* = 45°, the magnitude of phase shift |*δφ*| becomes 135° using Eq. (2) with a non-integer OAM value of *α* = 0.375 using Eq. (3).

2 $$|\delta \phi |=\delta \phi {N}_{ {\mathcal L} }$$

3 $$\alpha =\frac{|\delta \phi |}{2\pi l}$$

Thus, the generated beam by the $\frac{\pi }{2}$ three element aperture will possess a weak Quasi-OAM mode *α*. Since non-integer modes $\alpha \in {\mathbb{R}}$ are superpositions of orthogonal integer OAM modes $l\in {\mathbb{Z}}$, we can deduce the overall strength of the topological charge in a Quasi-OAM beam using Eq. (4) and its integral^24^ Eq. (5) as follows:

4 $${S}_{\alpha }=\mathop{\mathrm{lim}}\limits_{\rho \to \infty }\frac{Re}{2\pi }{\int }_{0}^{2\pi }d\varphi \,\frac{\sum _{-\infty }^{\infty }\,\frac{n}{n-\alpha }{P}_{n}(\rho )exp(in\,\varphi )}{\sum _{-\infty }^{\infty }\,\frac{1}{n-\alpha }{P}_{n}(\rho )exp(in\,\varphi )}$$

5 $${S}_{\alpha }=int(\alpha +\frac{1}{2})$$

We can see from Eq. (5) that the strength of non-integer OAM *α* falls to the nearest integer when $\alpha \ge \frac{1}{2}l$ (half-integer). Hence; the restriction on lower bounds of ${N}_{ {\mathcal L} }$ in previous work demonstrating Quasi OAM utilising a Quasi-Circular Array (QCA)^22^.Therefore, for small restricted apertures where the magnitude of the total phase shift |*δφ*| < *π*, the proposed weighted phase shift loading parameter *W*_*δφ*_ must be:

6 $${W}_{\delta \phi }\ge \frac{\pi l}{{N}_{ {\mathcal L} }}$$

This weighted phase shift parameter *W*_*δφ*_ in Eq. (6) is proposed to substitute the conventional phase shift parameter for annular apertures in Eq. (1), where the aperture is a restricted angular aperture less than *π*. Although the use of this weighted phase shift parameter is predicted to augment OAM modes produced by Quasi-Annular apertures >*π*, such as those fabricated by Fouda *et al*.^22^, and hence; improve mode cross-talk issues that are inherent to quasi annular apertures. However, the results in this paper will be confined to annular apertures <*π* to prove OAM augmentation feasible where aperture restriction is deemed impossible whilst adhering to the theoretical OAM phase shifts for mode stability.

In our numerical simulations and experiments, we take into account Eq. (5) and compare an OAM *α* = 0.375 (where *W*_*δφ*_ is not used) against the proposed phase weighted configuration. The phase weighted configuration is chosen to increase the total phase shift magnitude |*δφ*| to achieve a non-integer OAM *α* = 0.75 (where *W*_*δφ*_ = 90°), surpassing Berry’s theoretical derivation of $\frac{1}{2}l$ in Eq. (5) to increase intended *l* mode dominance, and satisfying Eq. (6).

**Figure 3:**
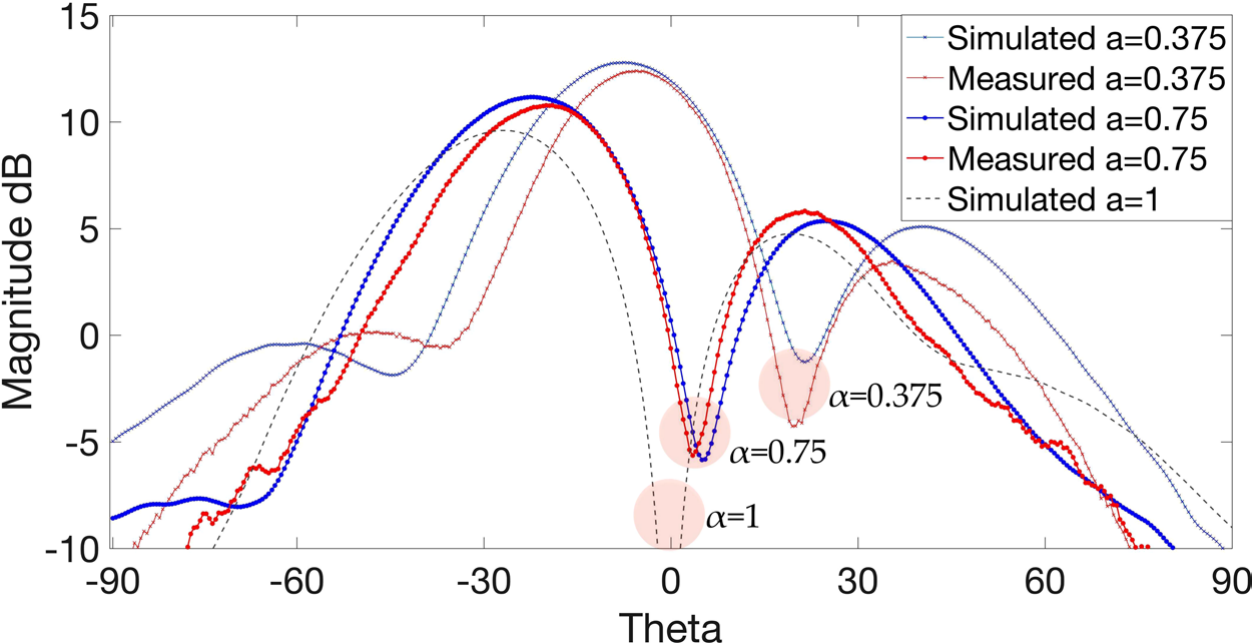
Measured and Simulated radiation patterns of *α* = 0.375 (*δφ* = 45°) and *α* = 0.75 (*W*_*δφ*_ = 90°). Simulated *α* = 1 (*W*_*δφ*_ = 120°) is displayed to show the vortex regions in the highlighted circles moving closer towards the *z* axis, as *α* becomes closer to integer *l*.

Simulated and experimental results of the radiation patterns for *α* = 0.375 and *α* = 0.75 are displayed in Fig. 3. Simulations were calculated on a plane 300 mm away from the aperture in the near-field, and measurements were taken 550 mm away from the aperture, in the far-field region, to measure mode stability. All measurements were conducted in an anechoic chamber, where each element is fed with a uniform amplitude and a corresponding phase shift using conventional Eq. (1) for *α* = 0.375 or the proposed Eq. (6) with a weighted phase shift for *α* = 0.75. What is interesting to note from the results shown in Fig. 3 is the movement of the main vortex towards the *z* axis in the boresight direction, as the magnitude |*α*| increases towards integer *l*. To further demonstrate this, we simulated a weighted phase shift parameter *W*_*δφ*_ = 120° to achieve *α* = 1 using Eq. (3) for OAM *l* = +1. As can be seen from Fig. 3, the weighted phase shift parameter *W*_*δφ*_ has relocated the vortex to the center position at *θ* = 0 where the OAM was augmented to *α* = 1. Therefore, as *α* deviates from integer mode *l*, the vortex tends to also deviate from the *z* axis.

**Figure 4:**
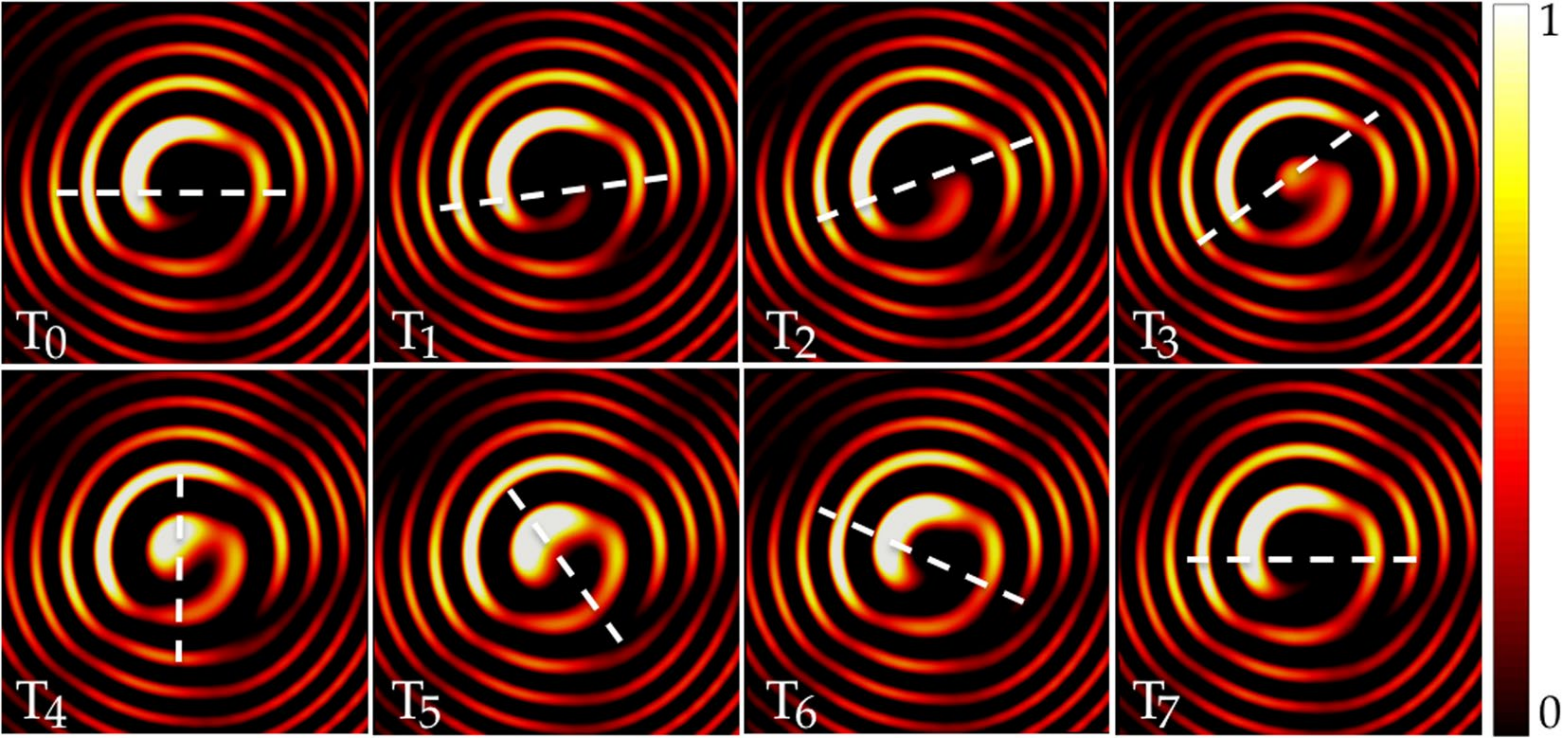
Rotational E-field intensity pattern of augmented OAM mode *α* = 0.75 using the weighted phase shift parameter *W*_*δφ*_ undergoing a 2*π* rotation around the propagation axis starting from time *T*_0_ to *T*_7_ (where *T*_7_ is the time it takes to complete a 2*π* revolution).

**Figure 5:**
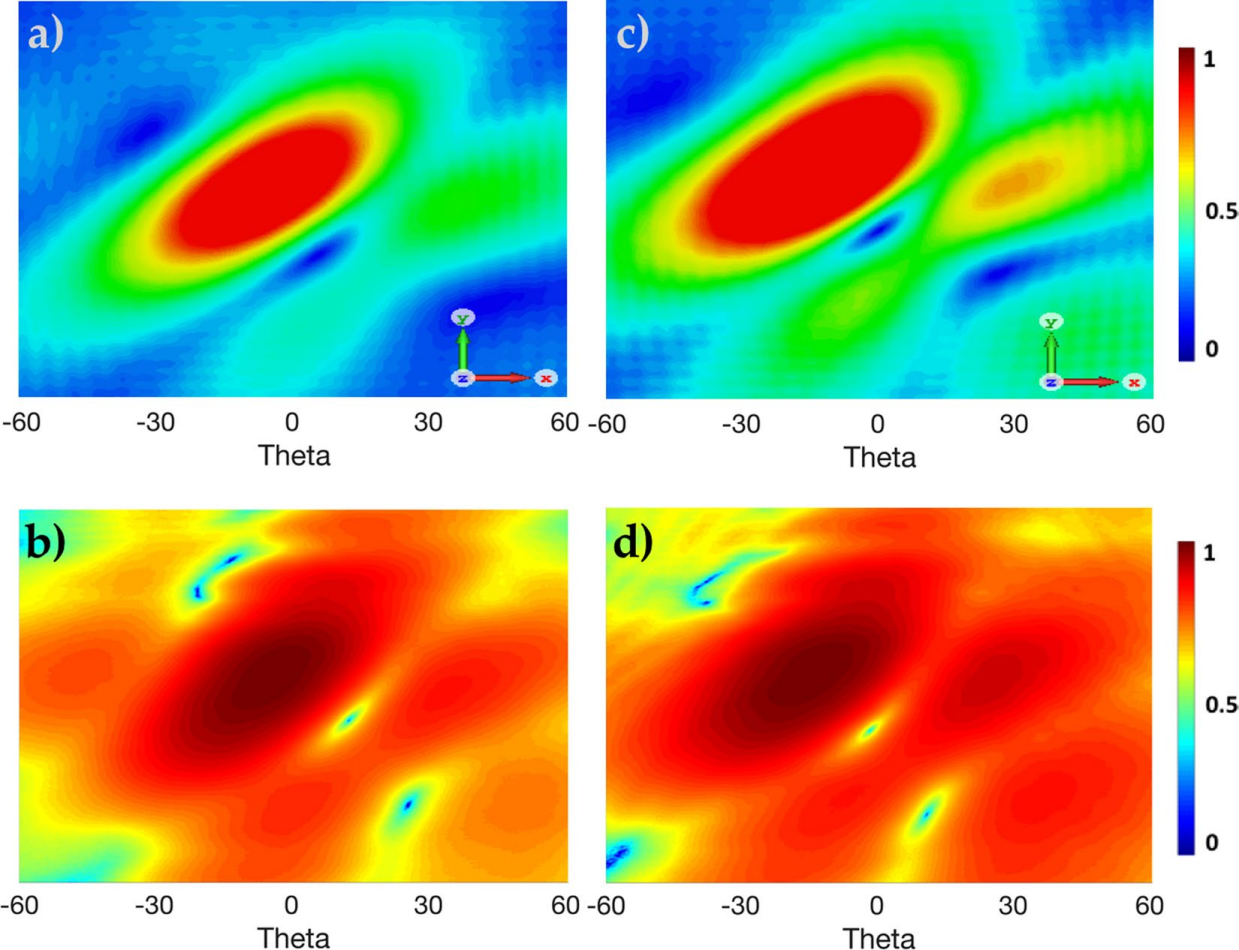
Simulated and Measured intensity patterns (**a**,**b**) for *α* = 0.375 and (**c**,**d**) for augmented *α* = 0.75 respectively.

**Figure 6:**
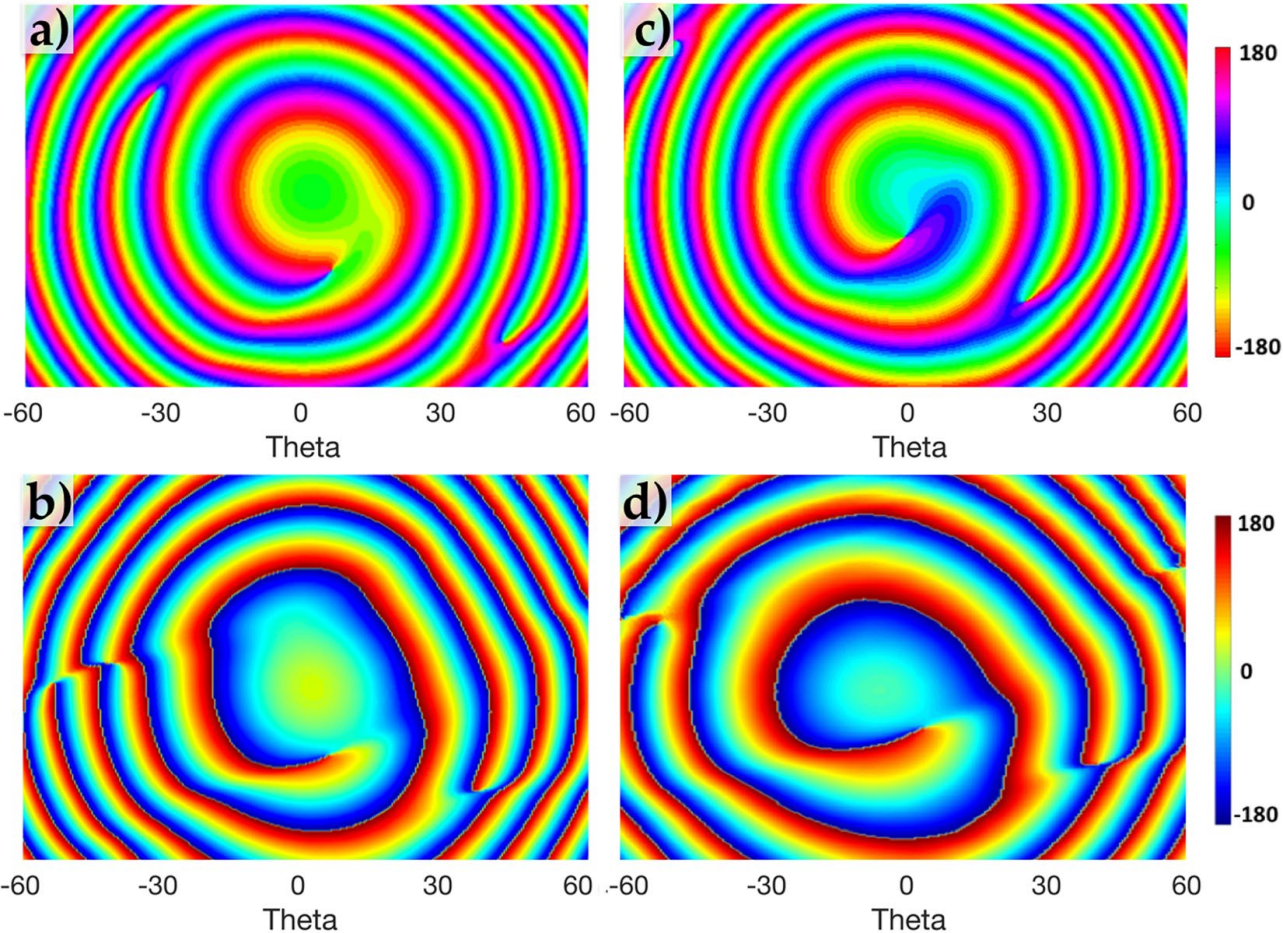
Simulated and Measured phase profile patterns (**a**,**b**) for *α* = 0.375 and (**c**,**d**) for augmented *α* = 0.75 respectively.

Figure 4 shows the rotational E-field intensity pattern of the augmented OAM mode *α* = 0.75 using the weighted phase shift parameter *W*_*δφ*_. It is evident that the rotation is an anticlockwise rotation (associated with dominant mode *l* = +1) starting from time *T*_0_ to *T*_7_ for a complete 2*π* rotation utilizing the momentum in the orbital angular momentum of a $\frac{\pi }{2}$ aperture. The simulated and measured intensity patterns of *α* = 0.375 and *α* = 0.75 are displayed in Fig. 5(a–d) respectively. Their corresponding simulated and measured phase profiles are shown in Fig. 6(a–d) respectively.

Both intensity and phase patterns of the augmented OAM *α* = 0.75 show the main vortex relocating towards the *z* axis and has a more prominent OAM *l* = +1 phase profile than *α* = 0.375. What is also interesting to note is the birth of another vortex in the near region of the *z* axis for both *α* = 0.375 and *α* = 0.75 as shown in Fig. 5(b,d). This confirms the theoretical predictions^21,24^ of other vortices born where *α* is a non-integer. This phenomena arises as non-integer vortices are not stable upon propagation, and eventually produce alternating *l* = ±1 vortices near the *z* region. As we augment *α*, the intensity of the beam around the second born vortex near *z* also increases, as can be seen in Fig. 5(d).

**Figure 7:**
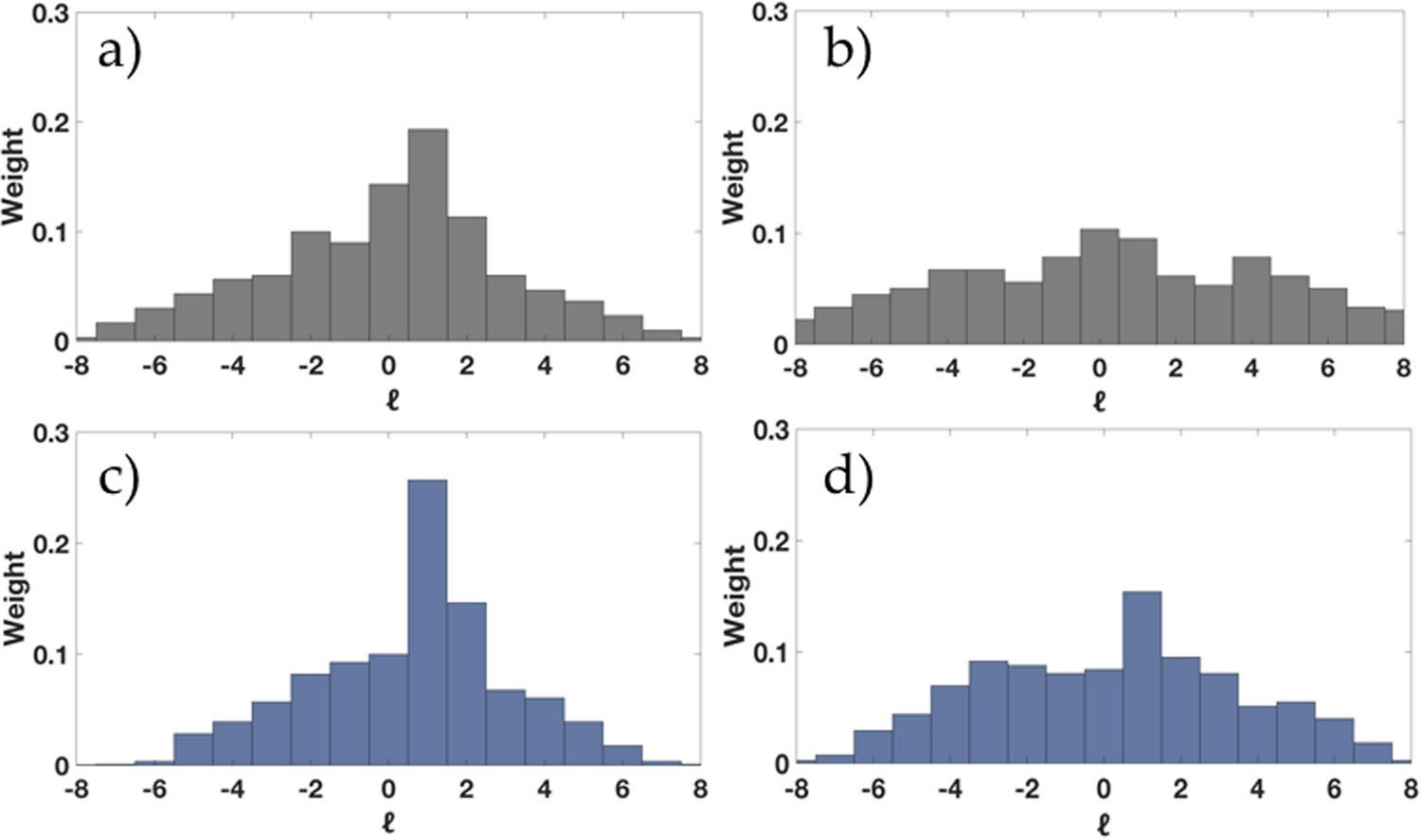
Simulated and Measured OAM spectrum weightings (**a**,**b**) for *α* = 0.375 and (**c**,**d**) for the proposed augmented *α* = 0.75 respectively.

As non-integer OAM *α* is a superposition of integer vortices *l*, the decomposition of OAM weightings can be derived using the Fourier relationship between OAM mode and angular position^20,27^. This Fourier relationship exists for all non-integer OAM modes, and has been previously decomposed within the Microwave region^28^ showing a clear distribution in angular momentum states. The simulated and measured OAM spectrum results in Fig. 7 show the relationship between the weighted phase shift parameter *W*_*δφ*_ and mode preciseness with reference to the uncertainty principle, as shown in Fig. 1. This is evident in both simulated and measured results, where the angular momentum distribution decreases upon augmenting |*α*| using the proposed weighted phase shift *W*_*δφ*_ parameter (Fig. 7(c,d)) as compared to a non-weighted phase shift in Fig. 7(a,b). Simulated and measured spectrum for *α* = 0.375 in Fig. 7(a,b) respectively, shows that the non-integer vortex being less than half-integer, is non-stable and falls back to the nearest integer *l* = 0 in the measurement far-field region. Augmented OAM *α* = 0.75 however, tends to remain dominant in the near-field and far-field regions as shown in Fig. 7(c,d) respectively, confirming Berry’s theoretical derivations in Eq. (5) and the validity of our proposed Eq. (6) for mode preciseness and stability upon propagation.

## Conclusion

In conclusion, a weighted phase shift parameter *W*_*δφ*_ is studied and proposed for less than *π* angular apertures to augment the OAM intended *l* mode to achieve mode stability upon propagation and preciseness in the near-field and far-field regions. A $\frac{\pi }{2}$ aperture consisting of three elements was used to demonstrate through simulations and experiments that augmenting the intended OAM *l* mode using the weighted phase shift method resulted in the main vortex relocating closer to the *z* axis as compared to a non-weighted phase shifted non-integer vortex. It was also shown that an augmented *α* = 0.75 OAM mode with a weighted shift parameter remained dominant in the far-field as opposed to *α* = 0.375 with a non-weighted phase shift falling back to *l* = 0 in the far-field as theoretically predicted. The OAM spectrum weightings were simulated and measured, indicating that the use of a weighted phase shift *W*_*δφ*_ resulted in a decrease in angular momentum distribution for the same $\frac{\pi }{2}$ aperture, hence; increased mode preciseness and dominance. The methods to augment OAM mode power described in this paper are also envisioned to improve OAM mode power produced by quasi apertures >*π* to improve inherent OAM mode cross-talk.

## Methods

Simulations of the proposed method to augment OAM mode power were conducted using CST Microwave Studio for electromagnetic waves and antenna simulations. The simulation results were fixed at the operating frequency of 10 GHz, and the complete design of the radiating elements and proposed QCA arrays were rendered in the simulation environment before fabrication. For the Q-OAM augmentation experiments conducted and showcased in this paper, each array is connected to a power divider by equal length phase-matched cables and phase shifters connected to each element in the array to achieve the phase configurations discussed in this paper. The feed signal was produced by an Anritsu MS4644B Vector Network Analyser (VNA) which was divided into *N* parts, where *N*_*Q*_ = 3 for the QCA of 3 elements. The probing antenna used to measure the output of the 3 element array aperture was a single back-launched horn antenna. This antenna was standardised against an ETS-Lindgren 3115 double ridged horn antenna. In the measurement setup, it was connected to an amplifier via the VNA. It was mounted on a scanning pylon and configured to cover 500 mm above boresight and 500 mm below in order to scan a 1 × 1 meter vertical plane. The measurements are taken in the far-field of the QCA based on the far-field criteria of *R*_*ff*_ = (2*D*^2^)/*λ*. The standardised antenna was positioned approximately 800 mm away from the arrays. All measurements were conducted in an anechoic chamber and at the operating frequency of 10 GHz.
